# Projections of human papillomavirus (HPV) vaccination impact in Ethiopia, India, Nigeria and Pakistan: a comparative modelling study

**DOI:** 10.1136/bmjgh-2021-006940

**Published:** 2021-11-01

**Authors:** Allison Portnoy, Kaja Abbas, Steven Sweet, Jane J Kim, Mark Jit

**Affiliations:** 1 Center for Health Decision Science, Harvard University T H Chan School of Public Health, Boston, Massachusetts, USA; 2 Department of Infectious Disease Epidemiology, London School of Hygiene & Tropical Medicine, London, UK; 3 Public Health Foundation of India, New Delhi, India; 4 Vitalant Research Institute, San Francisco, California, USA; 5 School of Public Health, The University of Hong Kong, Hong Kong, Hong Kong

**Keywords:** health policy, public health, vaccines, cancer

## Abstract

**Introduction:**

Cervical cancer is the second most common cancer among women in Ethiopia, India, Nigeria and Pakistan. Our study objective was to assess similarities and differences in vaccine-impact projections through comparative modelling analysis by independently estimating the potential health impact of human papillomavirus (HPV) vaccination.

**Methods:**

Using two widely published models (Harvard and Papillomavirus Rapid Interface for Modelling and Economics (PRIME)) to estimate HPV vaccination impact, we simulated a vaccination scenario of 90% annual coverage among 10 cohorts of 9-year-old girls from 2021 to 2030 in Ethiopia, India, Nigeria and Pakistan. We estimated potential health impact in terms of cervical cancer cases, deaths and disability-adjusted life years averted among vaccinated cohorts from the time of vaccination until 2100. We harmonised the two models by standardising input data to comparatively estimate HPV vaccination impact.

**Results:**

Prior to harmonising model assumptions, the range between PRIME and Harvard models for number of cervical cancer cases averted by HPV vaccination was: 262 000 to 2 70 000 in Ethiopia; 1 640 000 to 1 970 000 in India; 330 000 to 3 36 000 in Nigeria and 111 000 to 1 33 000 in Pakistan. When harmonising model assumptions, alignment on HPV type distribution significantly narrowed differences in vaccine-impact estimates.

**Conclusion:**

Despite model differences, the Harvard and PRIME models yielded similar vaccine-impact estimates. The main differences in estimates are due to variation in interpretation around data on cervical cancer attribution to HPV-16/18. As countries make progress towards WHO targets for cervical cancer elimination, continued explorations of underlying differences in model inputs, assumptions and results when examining cervical cancer prevention policy will be critical.

**Key questions**

**What is already known?**

- Studies have shown that prophylactic human papillomavirus (HPV) vaccination provides almost 100% protection against persistent infection with vaccine-targeted high-risk HPV strains (eg, HPV-16, 18) and associated precancers if administered prior to sexual initiation.
- Health-impact modelling has been used to project vaccination impact and estimate the timeline to eliminate cervical cancer as a public health problem (ie, reducing country-level annual cervical cancer incidence to below 4 per 100 000).

**What are the new findings?**

- The differences in outcomes between the models capture variation in interpretation around data on cervical cancer epidemiology and future demographic change.
- This study highlights that HPV-type distribution is a critical input to modelling the potential health impact of vaccination.

**What do the new findings imply?**

- In order to accelerate progress towards cervical cancer elimination, preventing cervical cancer through HPV vaccination will be an essential strategy, particularly given low coverage and access to cervical cancer screening in low-income and middle-income country settings.
- Understanding similarities and differences between HPV vaccination impact predicted by different models will be crucial, given that key questions about which countries to prioritise and which vaccination strategies to use will be important in an era of HPV vaccine dose shortages and COVID-19-related disruptions to vaccination programmes.

## Introduction

Persistent infections with human papillomavirus (HPV) types 16 and 18 cause 70% of all cases of cervical cancer.^1 2^ Studies have shown that prophylactic HPV vaccination provides almost 100% protection against persistent infection with vaccine-targeted high-risk HPV strains (eg, HPV-16, 18) and associated precancers if administered prior to sexual initiation.^3 4^ The WHO has set a goal to eliminate cervical cancer as a public health problem by 2100, which involves reducing country-level annual cervical cancer incidence to below 4 per 100 000.^5–7^ However, vaccine coverage remains low in low-income and middle-income countries (LMICs) that also lack high-quality screening programmes.^8^ More than 85% of cervical cancer deaths occur in LMICs^9^ with cervical cancer being the leading cause of female cancer death in sub-Saharan Africa.^10^

Cervical cancer imposes the second greatest burden of cancer incidence among women in Ethiopia, India, Nigeria and Pakistan as well as the second greatest burden of cancer mortality among women in Ethiopia, India and Nigeria—and the fourth greatest burden of cancer mortality among women in Pakistan.^11^ The International Agency for Research on Cancer (IARC) estimated that 148 000 new cases occurred and 94 000 women died from cervical cancer in these four countries in 2020.^11^ However, of these four countries, only Ethiopia has implemented nationwide HPV vaccination, with a single-age cohort campaign of 14-year-old girls in 2018–2019,^8 12^ while India has introduced it in a few states in 2016.^13^

Most new vaccine introductions in those countries have been supported through partnerships with the global community, particularly Gavi, the Vaccine Alliance, and the Bill and Melinda Gates Foundation (BMGF). Mathematical models of the impact of HPV vaccines have been used to support programme monitoring and priority setting by Gavi and BMGF since 2011.^14^ The Vaccine Impact Modelling Consortium (VIMC) was formed in late 2016, with the support of Gavi and BMGF, to bring together two groups involved in HPV modelling, with others conducting impact modelling of 11 other vaccines.^15^ Additionally, by ensuring that at least two groups model each analysed pathogen within VIMC, the vaccine-impact estimates from VIMC provide an important opportunity to examine the parametric, structural, model and methodological uncertainty both within and between models.^16^ Comparative modelling aims to enhance model transparency and can help guide public health research and priorities.

The two HPV vaccine models in VIMC are the Papillomavirus Rapid Interface for Modelling and Economics (hereafter PRIME), developed by a consortium of modellers led by the London School of Hygiene & Tropical Medicine and the Harvard model (hereafter Harvard), developed by a team of modellers at the Harvard T.H. Chan School of Public Health. Both models have been used extensively to inform decisions by Gavi,^17 18^ BMGF,^19^ WHO^20–22^ and individual countries.^23 24^ Given that both models are being used to understand HPV vaccine impact in the same settings, it is important to quantitatively compare the projections made by both models and to understand their differences, so that model results can be interpreted in the context of each other.

Our study objective was to assess similarities and differences in vaccine-impact projections through comparative modelling analysis by independently estimating the potential health impact of HPV vaccination among 10 cohorts of 9-year-old girls from 2021 to 2030 in Ethiopia, India, Nigeria and Pakistan. We used a vaccination scenario of 90% annual coverage among 9-year-old girls, in alignment with the goals of the cervical cancer elimination strategy set forth by the WHO.^5–7^ We estimated the potential health impact in terms of cervical cancer cases, deaths and disability-adjusted life years (DALYs) averted among vaccinated cohorts from the time of vaccination until 2100 in Ethiopia, India, Nigeria and Pakistan. We conducted a comparative modelling analysis to infer the differences in the vaccine-impact estimates generated by the PRIME and Harvard models.

## Methods

We used the PRIME and Harvard models to project the impact of HPV vaccination in four high-burden countries (Ethiopia, India, Nigeria and Pakistan). Both the PRIME^22 25^ and Harvard^17 23^ models have been extensively described and validated elsewhere; we summarise their main features below.

### Model overview

Both the Harvard and PRIME models are static, multicohort, proportional impact models that can estimate the impact of HPV vaccination on cervical cancer cases and deaths. The models estimate vaccination impact in terms of reductions in age-dependent cervical cancer incidence and mortality in direct proportion to vaccine efficacy against HPV-16/18, vaccine coverage and HPV-type distribution.

The models assume that girls are fully immunised with a two-dose schedule with perfect timeliness at the target ages and that girls effectively immunised against vaccine-targeted HPV types can develop cervical cancer associated with non-vaccine HPV types; also, neither cross-protection against non-vaccine types nor indirect effects are assumed. The models capture burden from all HPV genotypes, but the impact of vaccination is limited to the burden caused by genotypes targeted by the vaccine. In this analysis, the models simulated health benefits from vaccination against HPV types 16 and 18. Vaccine efficacy against HPV-16/18 infections is assumed to be 100%^26–30^ over the lifetime. Herd effects are not considered, so the vaccine-impact estimates produced are conservative. The models assume that age-specific cervical cancer incidence among unvaccinated women remains constant over the time horizon of the model.

### Data sources

Table 1 outlines the data sources used by the Harvard and PRIME models. Age-specific cervical cancer incidence is estimated from the database of IARC.^11^ For the proportion of cancer that is attributed to the vaccine-covered types (eg, HPV-16/18), PRIME uses a study by Serrano *et al*^31^ whose data sources include a meta-analysis performed by IARC^32^ and a retrospective cross-sectional worldwide study,^33^ while Harvard uses the meta-analysis by IARC^32^ exclusively.

**Table 1:** Data sources and overview of comparative analysis for Harvard and PRIME models

Feature	Harvard	PRIME
Model structure	Proportional outcomes	Proportional outcomes
Population representation	Open, multi-cohort	Open, multi-cohort
Representation of infection	Static	Static
Representation of cancer progression	Country-specific distributions of cancer stages, assuming 2 years lived with disability and 5 years survival for individuals experiencing cancer mortality^21^	Based on Global Burden of Disease-prescribed durations and phases (diagnosis and primary treatment phase, controlled phase, metastatic phase and terminal phase)
Disability-adjusted life year estimation	Based on weighted averages of Global Burden of Disease-prescribed weights for Stages I–III and Stage IV^34 35^	Based on Global Burden of Disease-prescribed weights^35^
Cervical cancer incidence	Globocan 2020 database of IARC^11^	Globocan 2020 database of IARC^11^
Cervical cancer mortality	Weighted averages of 5 year stage-specific survival probabilities for untreated and treated cervical cancers (by region) and treatment access proportions (by country)^11 21^	Globocan 2020 database of IARC^11^
Cervical cancer prevalence	Not applicable	Globocan 2020 database of IARC^11^
Population size	United Nations World Population Prospects 2019 estimates^36^	United Nations World Population Prospects 2019 estimates combined with time-varying, country-specific probability of death for projected estimates^36^
Life tables	World Health Organization 2019 life tables (constant)^37^	Constructed with United Nations Population Division time-varying, country-specific probability of death^36^
HPV-16/18 proportion	Meta-analysis by IARC^32^	Serrano *et al*^31^ based on meta-analysis by IARC^32^ and retrospective cross-sectional study^33^

HPV, human papillomavirus; IARC, International Agency for Research on Cancer; PRIME, Papillomavirus Rapid Interface for Modelling and Economics.

To estimate cancer mortality, the Harvard model assumes country-specific distributions of cancer stages.^21^ The model then incorporates 5-year stage-specific survival probabilities for untreated and treated cervical cancers (by region) and treatment access proportions (by country). These values are combined into weighted averages to provide country-specific 5-year survival parameters by stage, validated against age-specific mortality rates.^11 21^ The PRIME model uses estimates of age-specific cervical cancer mortality from Globocan 2020.^11^

In the Harvard model, disability weights are assumed to be 0.2 for stages I–III cervical cancer and 0.4733 for stage IV cervical cancer, based on the Global Burden of Disease (GBD) studies,^34 35^ and all cervical cancer cases experienced an average of 2 years lived with disability. In PRIME, disability weights are also based on GBD studies for the different phases of cervical cancer: diagnosis and primary treatment phase (0.288), controlled phase (0.049), metastatic phase (0.451) and terminal phase (0.540). The disability weights and durations for the different phases of cervical cancer are used in estimating the years of life lost due to disability.

### Demography

PRIME is a multiple cohort model. It calculates population size by estimating the size of the female age cohort at the age of vaccination (eg, 9 years old) from World Population Prospects 2019 estimates.^36^ The size of the age cohort in subsequent years is then calculated by constructing life tables, using the time-varying probability of dying by age and country from the World Population Prospects 2019 estimates.^36^

In contrast, the Harvard model is a population-based model that uses only data from the World Population Prospects 2019 estimates.^36^ The base year (eg, 2010) is used for the population projections in year 0, the next year (eg, 2011) is used for the population projections in year 1 and so forth. Life tables from the WHO are used for calculating DALYs, but not for population projections. Demographic estimates for age-specific population size (in 1-year intervals) and age-specific life expectancy (in 5-year intervals) were from United Nations World Population Prospects 2019 estimates^36^ and 2019 WHO life tables,^37^ respectively. In years when no data were available, we used a growth factor calculated as a function of a country’s population.

### Vaccination scenarios

We conducted analyses to evaluate the impact of HPV vaccination assuming 90% coverage of annual, routine vaccination of 9-year-old girls vaccinated in 2021–2030 (ie, ten cohorts). We assumed 100% protection against HPV-16 and 18 infections over the lifetime of vaccines for a two-dose vaccination schedule.

### Model outcomes

Cervical cancer cases, deaths and DALYs averted were calculated in comparison with a strategy of no HPV vaccination in the four high-burden countries (Ethiopia, India, Nigeria and Pakistan) using both PRIME and Harvard models. Model outcomes were aggregated over multiple birth cohorts to capture the health benefits of vaccinating girls aged 9 years between 2021 and 2030 from the time of vaccination until 2100.

### Comparative analysis

The principles of our comparative modelling study are: (1) addressing policy questions on HPV vaccination impact, (2) selection of two widely published models (Harvard and PRIME) to estimate HPV vaccination impact, (3) harmonisation of input data (demography, prevaccination cervical cancer burden and HPV 16/18-type distribution) and outputs (cervical cancer cases, deaths and DALYs), (4) exploring variability in estimates of HPV vaccination impact within and between Harvard and PRIME models, (5) presenting the comparative results and (6) projecting lifetime health impact among adolescent girls at 90% vaccination coverage (a key objective of the global cervical cancer elimination strategy).^38^ This aligns with the principles presented in the guidelines for multimodel comparisons of the impact of infectious disease interventions.^39^

Model inputs, including population demography and HPV-16/18-type distribution, were harmonised in order to evaluate model differences. Harmonisation refers to standardisation of input data and outputs between multiple models to address a research question.^39^ In our case, we standardised input data (demography, prevaccination cervical cancer burden, and HPV 16/18-type distribution) and outputs (cervical cancer cases, deaths and DALYs) in the Harvard and PRIME models to compare the projections in HPV vaccination impact (cases, deaths and DALYs averted by vaccination).

### Patient and public involvement

Patients and the public were not involved in this study.

## Results

### Descriptive model differences

**Figure 1:**
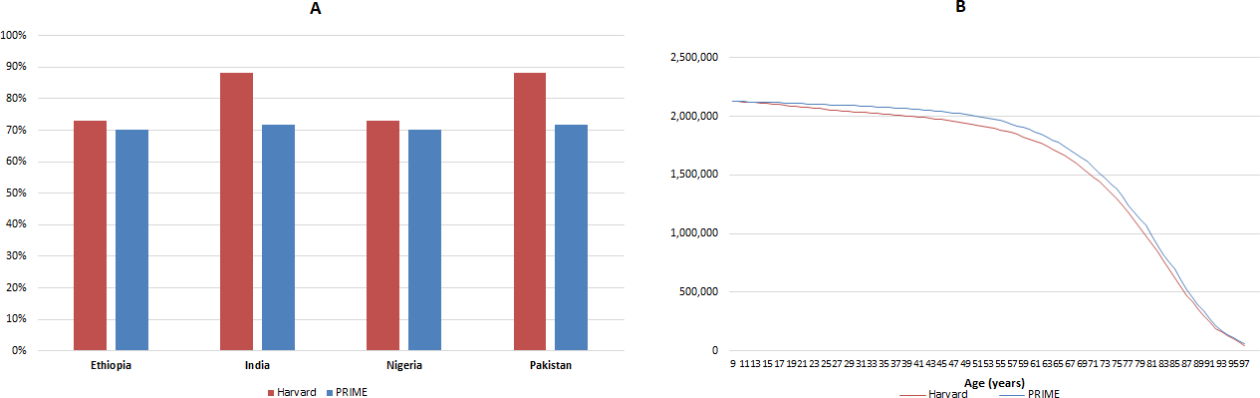
(A) Proportion of cervical cancer attributable to HPV-16/18; and (B) population size over time for 9-year-old girls born in 2012 (vaccinated in 2021) in Pakistan. HPV, human papillomavirus; PRIME, Papillomavirus Rapid Interface for Modelling and Economics.

Figure 1A shows the differences between the data used for the proportion of cervical cancer that is attributed to HPV-16/18 by the two models for Ethiopia, India, Nigeria and Pakistan. Figure 1B compares the cohort size for a cohort born in 2012 (which relates to the vaccinated cohort of 9-year-old girls in 2021) in the two models using Pakistan as an example; all four countries showed similar differences and trends. While, on average, the differences in cohort size range from 2% to 6%, the differences remain small until older ages (80 years and above), when the differences increased due to the decreasing population size with increasing all-cause mortality.

### Cervical cancer cases, deaths and DALYs averted

**Figure 2:**
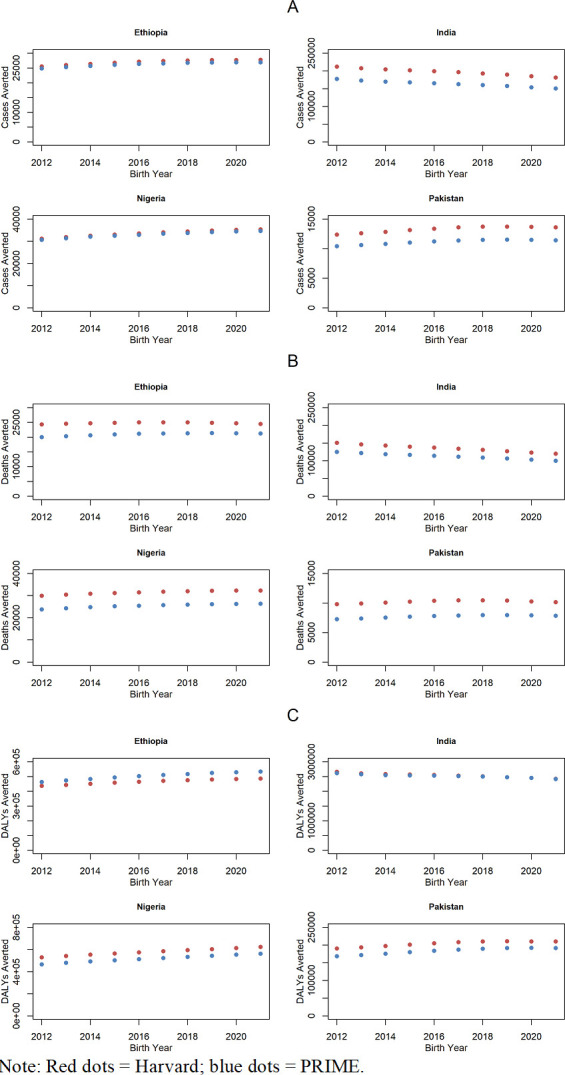
Cervical cancer cases, deaths and DALYs averted among girls vaccinated during 2021–2030 by country since time of vaccination until 2100: (A) cases averted; (B) deaths averted; (C) DALYs averted. DALYs, disability-adjusted life years; PRIME, Papillomavirus Rapid Interface for Modelling and Economics.

Under different assumptions for HPV-16/18-type distribution and demography, the Harvard model estimated a greater number of cervical cancer cases averted than the PRIME model by 3% in Ethiopia, 20% in India, 2% in Nigeria and 19% in Pakistan (figure 2A). Specifically, the range between the PRIME model and the Harvard model for the potential health impact of HPV vaccination in terms of the number of cervical cancer cases averted among girls vaccinated in 2021–2030 between the year of vaccination and 2100 was: 262 000 to 2 70 000 in Ethiopia; 1 640 000 to 1 970 000 in India; 330 000 to 3 36 000 in Nigeria and 111 000 to 1 33 000 in Pakistan.

Similarly, the Harvard model estimated a greater number of cervical cancer deaths averted than the PRIME model by 15% in Ethiopia, 17% in India, 19% in Nigeria and 24% in Pakistan (figure 2B). Specifically, the estimated number of cervical cancer deaths averted ranged from 210 000 to 2 48 000 in Ethiopia; 1 130 000 to 1 350 000 in India; 254 000 to 3 14 000 in Nigeria and 77 200 to 1 02 000 in Pakistan in the PRIME and Harvard models, respectively.

However, the PRIME model estimated a greater number of DALYs averted than the Harvard model by 8% in Ethiopia, whereas the Harvard model estimated a greater number of DALYs averted than the PRIME model by 1% in India, 11% in Nigeria and 10% in Pakistan (figure 2C). Specifically, the range between the PRIME model and the Harvard model for the estimated number of cervical cancer DALYs averted was: 4 650 000 to 5 030 000 in Ethiopia; 25 200 000 to 25 400 000 in India; 5 160 000 to 5 770 000 in Nigeria and 1 830 000 to 2 040 000 in Pakistan.

### Vaccination impact

**Figure 3:**
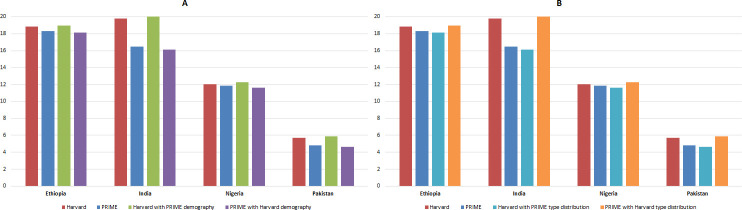
Cervical cancer cases averted per 1000 fully vaccinated girls for cohorts vaccinated during 2021–2030 since time of vaccination to 2100: (A) with alignment on population demography; and (B) with alignment on HPV 16/18-type distribution. HPV, human papillomavirus; PRIME, Papillomavirus Rapid Interface for Modelling and Economics.

Figure 3 shows the number of cervical cancer cases prevented per 1000 fully vaccinated girls in each of the four countries compared using both Harvard and PRIME models. When comparing the models under the respective model assumptions for HPV-type distribution and population demography, the estimated vaccination impact per 1000 fully vaccinated girls for the Harvard and PRIME models, respectively, was 19 versus 18 in Ethiopia; 20 versus 16 in India; 12 versus 12 in Nigeria and 6 versus 5 in Pakistan. When harmonising the assumptions around population demography across the Harvard and PRIME models, the difference in the estimated number of cervical cancer cases given routine vaccination narrowed, but the difference in the estimated vaccination impact was slightly increased (figure 3A). However, overall, the effect of harmonising the assumptions around population demography between Harvard and PRIME was small based on this metric, resulting in an equivalent estimate of the number of cervical cancer cases averted per 1000 fully vaccinated girls as the base case Harvard and PRIME models. On the other hand, when harmonising the assumptions around HPV-16/18-type distribution between the Harvard and PRIME models (figure 3B), the differences in estimated vaccination impact were nearly eliminated. Thereby, we infer that the main difference in estimates for cases averted by vaccination between the two models is due to variations in cervical cancer attribution to HPV-16/18.

## Discussion

The Harvard and PRIME models are used by both VIMC and other global stakeholders to project the impact of HPV vaccination. The two models differ in their inputs and assumptions for HPV-16/18-type distribution, population demography, cervical cancer mortality and estimation of DALYs. The proportion of cervical cancers due to HPV-16/18 is relatively higher in the Harvard model, especially for India and Pakistan, and thereby cases averted (figure 2A) and vaccination impact (cases averted per 1000 fully vaccinated girls) are relatively higher (figure 3) in the Harvard model for India and Pakistan. The difference between these two models captures variation around interpretation of input data. In the case of HPV-16/18-type distribution, the Harvard model relied on a meta-analysis of cross-sectional high-risk HPV-type distribution in HPV-positive women,^32^ whereas the PRIME model relied on^32^ the study by Serrano et al.^31^ whose data sources included the same meta-analysis^32^ and a retrospective cross-sectional worldwide study,^33^ and accounts for multitype infections through proportional weighting attribution.^31^

HPV-16/18 vaccination was estimated to avert substantial numbers of cervical cancer cases, deaths and DALYs by both the Harvard model and the PRIME model. Both models provide similar results to previous analyses of vaccination impact in the analysed countries, including multicountry^14 15 17–21 40 41^ and single-country analyses,^12 42^ most of which used earlier versions of the Harvard and PRIME models.

However, routine HPV vaccination has yet to be introduced in any of these four high-burden countries at the national level (although a nationwide single-age cohort campaign of 14-year-old girls was conducted in Ethiopia in 2018–2019^12^ and India introduced in a few states in 2016^13^). In order to accelerate progress towards cervical cancer elimination, preventing cervical cancer through HPV vaccination will be an essential strategy in these countries, particularly given the low coverage and access to cervical cancer screening.^8^ Understanding similarities and differences between HPV vaccination impact predicted by different models will be crucial, given that key questions about which countries to prioritise and which vaccination strategies to use will be important in an era of HPV vaccine dose shortages^43^ and COVID-19-related disruptions to vaccination programmes.^44 45^

The size of the relevant population at each year of age is critical because it determines the number of people who are exposed to the risk of cervical cancer and can, therefore, be protected by vaccination. Both methods used to estimate the at-risk population have strengths and limitations. In the PRIME model, the size of the age cohort in subsequent years is calculated by constructing life tables, using the time-varying probability of dying by age and country from the World Population Prospects 2019 estimates.^36^ The Harvard method not only captures such changes but also includes future population change due to migration, which may not reflect the same vaccination status as the locally born population. In general, as the demography differences are minimal between the two models, the vaccination-impact estimates are similar for each country when demography is switched to align with the alternative model. The main driver of the differences in the vaccination impact was HPV-16/18-type distribution—or, the proportion of cervical cancers that can be averted by the bivalent vaccine, which has a direct relationship with vaccination impact. As cervical cancer deaths are directly estimated from cervical cancer cases, HPV-16/18-type distribution is likewise the main driver of the differences in vaccination impact in terms of cervical cancer deaths averted, with additional differences due to the mortality estimation approach: stage-specific (Harvard) versus age-specific (PRIME). The years lived with disability for the estimated cervical cancer cases and the years of life lost for the estimated cervical cancer deaths both contribute to the estimates of DALYs averted by HPV vaccine, and, therefore, are likewise driven by differences in HPV-16/18-type distribution. However, the disability weights assumed by the Harvard and PRIME models differ and contribute to additional differences.

This comparative modelling exercise highlighted differences in the estimates of health impact of HPV vaccination due to model uncertainty. We note that HPV vaccine projections are particularly susceptible to large swings in estimated health outcomes due to even small changes in fertility and mortality because of the long time horizons needed in the models. Comparative modelling exercises as we have done can enhance model transparency and clarify the range of uncertainty in vaccine impact. Hence, the differences between the models are a strength that reflects the variation and uncertainty in the projected health outcomes of vaccination impact. Understanding the intermodel variation improves the quality and coordination of vaccine-impact assessment, which in turn can help guide public health research and priorities in cervical cancer elimination and control.

Similar comparative modelling exercises were conducted to examine the timeline to cervical cancer elimination in LMICs.^20 21^ Relying on evidence synthesis from different models was deemed an essential aspect to inform strategies for cervical cancer elimination by the WHO.^5 38^ However, these analyses relied on estimations of age-standardised cervical cancer incidence, such that demographic changes were not expected to drive the uncertainty in the timing of elimination.

There are several important limitations to this analysis. As we relied on static cohort models in these analyses, we were only able to estimate direct effects for vaccinated women, which excluded additional indirect benefits from herd immunity for unvaccinated women. We projected intervention impact for only 10 cohorts of 9-year-old girls in four countries and assumed that cervical cancer incidence rates affecting these cohorts would be stable over the time period of the analysis. Vaccine efficacy against high-risk HPV types other than HPV-16/18 (ie, cross-protection) was not included. We did not examine cervical cancer screening programmes in this analysis and assumed that any ongoing screening programmes did not change as HPV vaccination introduction and delivery changed. Additionally, given limited data on the burden of other HPV-related diseases in LMICs, we did not evaluate the impact HPV vaccination may have on non-cervical cancers in women and men.

## Conclusion

Both models project that HPV vaccination will have a large impact on morbidity and mortality in the four countries we examined. The differences in outcomes between the models capture variation in interpretation around data on cervical cancer epidemiology and future demographic change. This study highlights that HPV-type distribution is a critical input to modelling the potential health impact of vaccination. The main difference in estimates for cases and deaths averted by vaccination between the models capture variation in interpretation around data on cervical cancer attribution to HPV-16/18. The main differences in estimates for DALYs averted by vaccination between the models are due to variations in cervical cancer attribution to HPV-16/18, disability weights, and age-specific life expectancy. Continued explorations of underlying differences in model inputs, assumptions, and results will be crucial when examining public health policy.

## Data Availability

Data are available upon reasonable request. The datasets generated during and/or analysed during the current study are available from the corresponding author on reasonable request.
